# Breast Cancer Plasticity after Chemotherapy Highlights the Need for Re-Evaluation of Subtyping in Residual Cancer and Metastatic Tissues

**DOI:** 10.3390/ijms25116054

**Published:** 2024-05-31

**Authors:** Irena Barbara Padzińska-Pruszyńska, Muhammad Waqas Akbar, Murat Isbilen, Emilia Górka, Baris Kucukkaraduman, Seçil Demirkol Canlı, Ege Dedeoğlu, Shila Azizolli, Isli Cela, Abbas Guven Akcay, Hasim Hakanoglu, Lubomir Bodnar, Szczepan Cierniak, Zygmunt Kozielec, Jacek Jerzy Pruszyński, Martyna Bittel, Ali Osmay Gure, Magdalena Król, Bartłomiej Taciak

**Affiliations:** 1 Center of Cellular Immunotherapies, Warsaw University of Life Sciences, 02-786 Warsaw, Poland; irena_pruszynska1@sggw.edu.pl (I.B.P.-P.); magdalena_krol@sggw.edu.pl (M.K.); 2 Department of Molecular Biology and Genetics, Bilkent University, Ankara 06800, Turkey; muhammad.akbar@yale.edu (M.W.A.); guven.akcay@bric.ku.dk (A.G.A.); hhakanoglu@agcenter.lsu.edu (H.H.); 3 Department of Biostatistics and Bioinformatics, Acibadem University, Istanbul 34752, Turkey; 4 Molecular Pathology Application and Research Center, Hacettepe University, Ankara 06100, Turkey; 5 Department of Genetics and Bioengineering, Istanbul Bilgi University, Istanbul 34060, Turkey; 6 Department of Clinical Oncology and Radiotherapy, St. John Paul II Mazovia Regional Hospital in Siedlce, 08-110 Siedlce, Poland; 7 Faculty of Medical and Health Sciences, University of Natural Sciences and Humanities, 08-110 Siedlce, Poland; 8 Department of Pathomorphology, Military Institute of Medicine, 04-141 Warsaw, Poland; 9 Department of Pathomorphology, Warmian-Masurian Cancer Center of the Ministry of the Interior and Administration’s Hospital, 11-041 Olsztyn, Poland; 10 Department of Pathomorphology, University of Warmia and Mazury, 10-719 Olsztyn, Poland; 11 Department of Geriatrics and Gerontology, School of Public Health, Centre of Postgraduate Medical Education, 02-673 Warsaw, Poland; 12 Skopos Global Ltd., Atasehir, Istanbul 34750, Turkey; ali.gure@skoposglobal.com

**Keywords:** TNBC, breast cancer, prognostic markers, PAM50 plasticity, WNT/Mes

## Abstract

This research paper presents a novel approach to identifying biomarkers that can be used to prognosticate patients with triple-negative breast cancer (TNBC) eligible for neoadjuvant therapy. The study utilized survival and RNA sequencing data from a cohort of TNBC patients and identified 276 genes whose expression was related to survival in such patients. The gene expression data were then used to classify patients into two major groups based on the presence or absence of Wingless/Integrated-pathway (Wnt-pathway) and mesenchymal (Mes) markers (Wnt/Mes). Patients with a low expression of Wnt/Mes-related genes had a favorable outcome, with no deaths observed during follow-up, while patients with a high expression of Wnt/Mes genes had a higher mortality rate of 50% within 19 months. The identified gene list could be validated and potentially used to shape treatment options for TNBC patients eligible for neoadjuvant therapy providing valuable insights into the development of more effective treatments for TNBC. Our data also showed significant variation in gene expression profiles before and after chemotherapy, with most tumors switching to a more mesenchymal/stem cell-like profile. To verify this observation, we performed an in silico analysis to classify breast cancer tumors in Prediction Analysis of Microarray 50 (PAM50) molecular classes before treatment and after treatment using gene expression data. Our findings demonstrate that following drug intervention and metastasis, certain tumors undergo a transition to alternative subtypes, resulting in diminished therapeutic efficacy. This underscores the necessity for reevaluation of patients who have experienced relapse or metastasis post-chemotherapy, with a focus on molecular subtyping. Tailoring treatment strategies based on these refined subtypes is imperative to optimize therapeutic outcomes for affected individuals.

## 1. Introduction

Breast cancer stands as the most prevalent cancer among adults worldwide, with over 2.3 million reported cases annually [1]. Despite advances in treatment, some subtypes of breast cancer still remain a leading cause of cancer-related mortality, particularly affecting women. Alarmingly, nearly 80% of breast and cervical cancer deaths occur in low- and middle-income countries, highlighting significant global disparities [2].

Breast cancer (BC) is a complex disease encompassing various molecular subtypes, each with distinct characteristics, outcomes, and therapeutic considerations. Current clinical practice categorizes breast cancer into three subtypes based on immunohistochemistry (IHC) tests for estrogen receptor (ER), progesterone receptor (PR), and erythroblastic oncogene B/human epidermal growth factor receptor 2 (ErbB2/HER2) expression. While this approach guides treatment decisions, it may not fully capture the genetic diversity of tumors or accurately predict treatment response [3]. Genome-wide sequencing and gene expression profile analyses have demonstrated Wnt signaling to be an important player involved mainly in the processes of breast cancer proliferation and metastasis [4]. Tumor metastasis is a multistep process involving tumor cells disseminating from their primary site and migrating to the secondary organ. Epithelial–mesenchymal transition (EMT) is one of the crucial steps that initiates this process. The regulation of EMT is centered by several signaling pathways, including Wnt signaling [5]. Triple-negative breast cancer (TNBC), lacking the expression of ER, PR, and ErbB2/HER2, presents the poorest prognosis among breast cancer subtypes, with chemotherapy as standard treatment. However, response rates to chemotherapy are suboptimal, necessitating better patient stratification for treatment decisions. Recent efforts have aimed to subclassify TNBC based on biomarker expression patterns, with the potential to improve treatment selection and outcomes [6]. The survival analysis demonstrated that the current IHC-based classification could mislead the treatment and therefore result in worse outcome. Current guidelines for IHC might be updated accordingly [7].

Our research builds on previous success in identifying prognostic gene signatures across various cancer types, including pancreatic [8], colon [9], gastric [10], and breast cancers [11]. In this study, we aim to identify a gene list to prognosticate TNBC patients eligible for neoadjuvant therapy, a critical step toward personalized treatment strategies. Our novel approach by leveraging RNA sequencing and TCGA data, we identified 276 genes associated with patient survival, allowing classification into two distinct groups with differing outcomes.

Furthermore, our analysis of pre- and post-chemotherapy tumor samples revealed significant changes in gene expression profiles, indicating the dynamic nature of TNBC following treatment. We also investigated subtype switches between primary and metastatic tumors, providing insights into tumor evolution and potential implications for treatment selection.

The primary significance of our work lies in providing preliminary data for subsequent validation on larger datasets, with the aim of refining the identified gene list for personalized treatment approaches in TNBC patients eligible for neoadjuvant therapy. By enhancing patient outcomes and guiding treatment decisions, our research contributes to the ongoing efforts to improve TNBC management and develop more effective therapeutic interventions.

In summary, our findings underscore the importance of tailored treatment modalities for TNBC patients undergoing neoadjuvant therapy, highlighting the potential to significantly impact patient prognosis and advance TNBC treatment strategies.

## 2. Results

### 2.1. Identifying a Prognostic Signature for TNBC Patients

**Figure 1:**
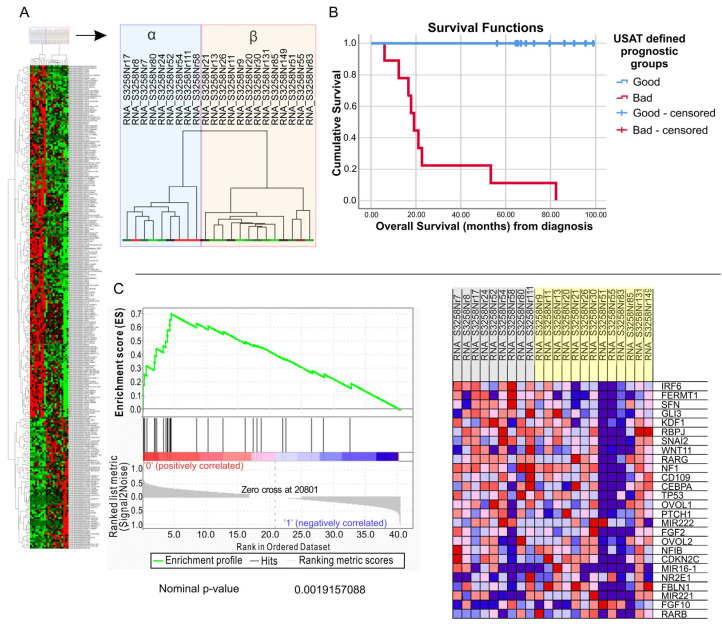
Neo-P gene list identifies two groups with clear prognostic and distinct biological features. Hierarchical clustering analysis with all 276 genes in Neo-P reveals two clear clusters: α and β (**A**); green: low gene expression; red: high gene expression. Kaplan–Meier plot showing patients with bad (**A**) and good (**B**) prognosis. Log-rank *p* < 0.0001 (**B**). Gene set enrichment analysis of prognostic groups identified by Neo-P reveal tendency towards enrichment of the “negative regulation of stem cell and proliferation” (GO number: 2000647) gene set in samples from patients with worse prognosis (**C**).

RNA sequencing data from 22 TNBC patients who received neoadjuvant chemotherapy and had samples obtained prior to therapy were used for Unsupervised Survival Analysis Tool (USAT) analysis. The 276 genes which had significant (<0.05) *p* values in all three tests were selected to generate a gene list (Neo-P). Additional data for these 276 signature genes are provided in the Supplementary Materials (Supplementary Table S1). When Neo-P was used to cluster the samples, two clear groups (A and B) with differential gene expression were visible (Figure 1A). When these groups were analyzed for survival differences, group B samples were observed to have a very good survival (no patients died within the observation period), whereas group A samples had a much worse prognosis, with a median survival time of 19 months, with the difference being significant (*p* < 0.001, log-rank test) (Figure 1B). When we performed gene set enrichment analyses to determine functional groups within these genes, we found a “negative regulation of stem cell and proliferation” gene set showing a tendency towards enrichment in patients with a worse prognosis (Figure 1C). The results were not statistically significant in all tests (nominal *p*-value 0.001, FDR q-value 1.0, FWER *p*-value 0.509). A table with significant gene sets is included in the Supplementary Materials (Supplementary Table S2).

To determine if Neo-P was a prognostic indicator independent of other clinical determinants of prognosis, we first performed univariate survival analyses (OS, from the date of diagnosis) on patients with pre-treatment samples and who received adjuvant chemotherapy (Figure 1C). According to these analyses, progression status, radiological regression, clinical regression, and relapse were significant (*p* < 0.05) prognostic factors (Supplementary Table S3). However, tumor quadrant, tumor localization: left/right, biopsy type, grade, pathological response, and The American Joint Committee on Cancer (AJCC) stage were not. Similarly, T stage and N stage, by themselves, were also not significant prognostic indicators in this cohort (Supplementary Table S4). Some of these parameters (progression status, radiological regression, clinical regression and relapse state) were significantly correlated (Supplementary Table S5). A multivariate analysis for patients with these four parameters identified either relapse (Backward Wald) or clinical regression (Forward Wald) as a single independent prognostic factor (Supplementary Table S6). We then analyzed USAT-defined prognostic groups (identified by Neo-P) with clinical regression in a multivariate model. This showed that the two did not identify separate patients; i.e., they were not independent of each other (Supplementary Table S7). Therefore, Neo-P identifies patients with clinical regression in this cohort of neoadjuvant treatment-receiving patients. We then used Neo-P to cluster three other datasets (all microarray: GSE2034, GSE11121 and GSE28844). GSE28844 is a dataset containing neoadjuvant (paclitaxel) treatment-receiving patients, from which we selected pre-chemotherapy samples. However, in none of these cohorts could Neo-P identify specific sub-groups (Supplementary Figure S1). As this analysis could serve as an in silico validation of Neo-P’s ability to determine prognostic groups, we performed further analyses as detailed below.

### 2.2. Validating a Previous Signature (Non-Stem Cell-like/EPITHELIAL (NS/E) vs. Stem Cell-like/Mesenchymal (CS/M)) in Our Patient Cohort

**Figure 2:**
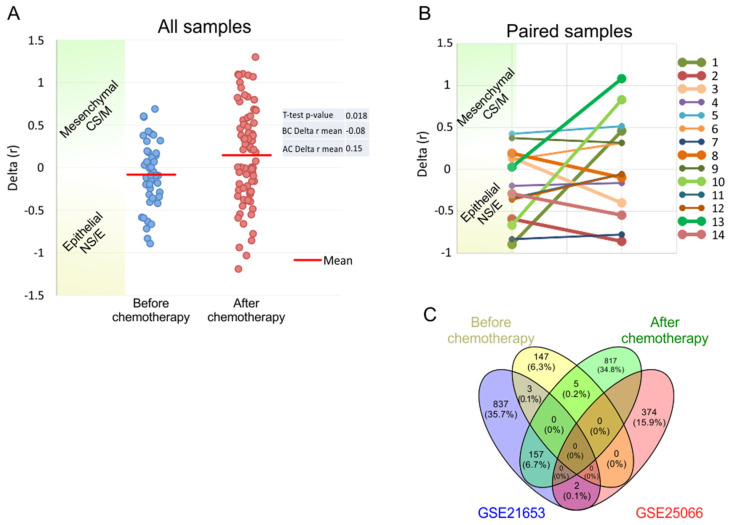
Gene expression differences among pre- and post-chemotherapy patient samples. Stemness scores calculated for all samples (**A**) and paired tumor samples (**B**), showing an increase in stemness and mesenchymal features post-chemotherapy. Before chemotherapy samples: 43; after chemotherapy samples: 78. *T*-test *p* values are 0.02 and 0.1, respectively. Very limited overlap of USAT identified genes from pre- and post-therapy patient samples, as well as from two other datasets (GSE21653 and GSE25066), showing a dramatic difference in gene expression induced by therapy (**C**).

Previously, we developed and published a gene signature (non-stem cell-like/epithelial (NS/E) vs. stem cell-like/mesenchymal (CS/M)), which we showed to have drug sensitivity predictive power, but which was not valuable from a prognosis determination point of view [11]. To test if this observation could be reproduced in this cohort, we compared samples from patients before and after chemotherapy. As can be seen in Figure 2A, when the correlation values of each sample to NS/E and CS/M were calculated and their difference taken (Delta (r)), a shift from epithelial to mesenchymal could be observed in our cohort, following chemotherapy (*p* < 0.02, *t*-test).

We then compared matched samples in the same way. As can be seen in Figure 2B, for most matched samples, there was a shift to a more mesenchymal state. Although *t*-test significance here was not observed (*p* = 0.1), due to the small number of samples, as the mean difference between the two groups is larger (0.25) when compared to all samples (0.23). The exact change in the tumor characteristics of those patients was described by Akbar et al. [11]. This shows that chemotherapy before surgery has a tendency to shift tumor gene expression to a more mesenchymal/stem cell-like phenotype, which is known to be related to drug resistance.

When we analyzed patients from our cohort according to USAT, first separating them into “before chemotherapy” (BC) and “after chemotherapy” (AC) groups, we found that genes that could predict prognosis in the two groups did not overlap (Figure 2C). This shows that these two tumor groups are phenotypically different and supports the observation that chemotherapy treatment changes the tumor phenotype to a degree that genes that can predict prognosis are no longer valid after chemotherapy. The figure shows that USAT results generated independently from two other cohorts do not generate overlapping genes either. This could also support the idea that the chemotherapy treatment status of tumors is critical for correct prognostic gene identification.

### 2.3. Correlating qPCR to RNAseq Data

To determine a gene list which could predict chemotherapy sensitivity to either 5-FU, Docetaxel or Paclitaxel, we analyzed three cell line/drug testing databases (GDSC1, GDSC2 and CTD) for genes whose expression correlated with sensitivity to either of these drugs. The correlation of these variables was assessed using Pearson correlation. Only 51 genes (0.1% of all genes with significant correlations) were found to be commonly related to drug sensitivity in all three databases (Supplementary Figure S2). Among these, 17 were selected for qPCR experiments. Of these 17 genes, three (JMJD7, LIN9 and TAL2) showed a correlation between increased gene expression and increased sensitivity to drugs, whereas the other 14 genes showed increased expression related to resistance to drugs as tested by Spearman analyses (Table 1, columns 2–4).

We then correlated the mean expression obtained by qPCR for all these genes in samples before and after chemotherapy. Among the 17 genes analyzed by qPCR, 13 genes showed expression differences between pre- and post-therapy samples in the same direction as expected (highlighted in blue, column 5). We then compared RNAseq data obtained from the same samples, before and after chemotherapy, and asked if the general change in expression was similar to what was obtained by qPCR. When RNASeq data were normalized based on DESeq, among 17 genes, 12 showed expression changes in the same direction as qPCR results (green highlight, column 9), whereas if RNASeq data were normalized as Fragments Per Kilobase Million (FPKM) or Trimmed Mean of *M*-values (TMM), these numbers were 4 and 5, respectively (Table 1).

### 2.4. PAM Plasticity

To explore the plasticity of PAM50 classes and understand how tumor subtypes may switch upon drug treatment, we conducted a PAM50 plasticity analysis in this manuscript. Plasticity refers to the ability of tumor subtypes to change, potentially influenced by processes such as epithelial–mesenchymal transition (EMT) and changes in immunological status.

In our analysis, we examined different datasets containing matched tumor samples with gene expression data available both before and after treatment. For instance, in the comparison of Letrozole pre- and post-treated samples from the GSE10281 dataset, we observed significant PAM50 class plasticity. Specifically, 39% of samples (7 out of 18) maintained their PAM50 class before and after Letrozole therapy, while 61% (11 out of 18) underwent a change in class.

Among the samples that retained their PAM50 class, basal subtype (n = 2) and LumB subtype (n = 1) were the most stable. Conversely, among the samples that experienced a change in subtype, a variety of transitions were observed. For example, one HER2, two LumA, and one normal subtype samples retained their original subtype, while three LumA, three HER2, and four normal subtype samples switched to another subtype.

Notably, the majority of samples that underwent subtype switching (n = 6) transitioned to the LumB subtype, while four samples switched to the basal subtype (Table 2). This analysis provides valuable insights into the dynamic nature of tumor subtypes in response to treatment, shedding light on potential mechanisms underlying treatment resistance and informing future therapeutic strategies.

Among patients treated with anthracycline- and taxane-based neoadjuvant chemotherapy (GSE32518), two basal samples, one HER2, three LumA, three LumB and one normal retained their subtype and two basal, three HER2, four LumA, eight LumB and one normal switched their phenotype (Table 3). Most samples switched their subtype to normal-like tumors.

In another dataset (GSE32072), gene expression data for 21 cancer samples were available for pre- and post-neoadjuvant therapy (nine patients receiving anthracycline-based therapy, eight patients receiving anthracycline/taxane-based therapy and two patients receiving trastuzumab therapy). Additionally, four patients were also included which were not treated with any neoadjuvant therapy as control. Upon PAM50 subtyping, patients who did not receive any neoadjuvant chemotherapy, retained their classes (two basal and two LumA). Interestingly, all basal (n = 5), Her2 (n = 4) and LumB (1) retained their subtype. Out of six pre-therapy LumA samples, only one changed to LumB subtype. Normal subtype samples changed the most as compared to other subtypes in this dataset (n = 4) and only one sample retained its phenotype (Table 4).

### 2.5. Identifying Metastatic Markers

Additionally, breast cancer metastatic samples can switch to another PAM50 class when compared with primary tissue class. In dataset GSE125989, 16 matched tissues were available for both brain metastasis and primary site. These samples were PAM50 subtyped. Overall, 50% (eight) of them retained their phenotype when primary site vs. metastasis PAM50 classes were compared. One basal changed to Her2 and one Her2 changed to basal. Major subtype switching was observed in the normal primary tissue phenotype, where three metastasized tissues became basal and two became HER2 (Table 5).

Dataset GSE110590 contains gene expression for 16 primary breast cancer tissues and their 67 matched metastases. Overall, 55% (37/67) of metastatic tissues retained the same PAM50 class when compared with the primary site. The most stable classes were basal where 85% (23/27) metastatic sites retained basal phenotype and HER2 100% (4/4), while LumA showed 32% (10/31) and normal showed 0% (0/5) consistency. Most primary samples when metastasized switched to basal and LumB class (Table 6). The statistical analysis for Table 2, Table 3, Table 4, Table 5 and Table 6 is included in Supplementary Material (Supplementary Table S8).

## 3. Discussion

The study presents a comprehensive analysis of triple-negative breast cancer (TNBC), focusing on the prognostic significance of a novel gene list (Neo-P) derived from RNA sequencing data of TNBC patients undergoing neoadjuvant chemotherapy. Through a Unified Score-based Association Test (USAT) analysis, Neo-P effectively stratified patients into two distinct groups, revealing significant differences in clinical outcomes. Notably, patients in Group B demonstrated markedly improved survival rates compared to those in Group A, underscoring the potential of Neo-P as a valuable prognostic indicator for TNBC patients undergoing neoadjuvant therapy.

Moreover, gene set enrichment analyses provided functional insights into the molecular mechanisms underlying treatment resistance and poor outcomes in TNBC patients. The demonstrated tendency towards enrichment of genes involved in negative regulation of stem cell and proliferation pathways in patients with a worse prognosis highlights the importance of understanding the molecular drivers of aggressive disease phenotypes and requires further research on a larger patient population [12,13]. These findings offer potential therapeutic targets and pathways for intervention to improve treatment responses and patient outcomes in TNBC.

In evaluating the independent prognostic value of Neo-P compared to other clinical determinants, Neo-P was found to be associated with clinical regression, a significant prognostic factor in TNBC patients receiving adjuvant chemotherapy. This suggests that Neo-P may complement existing clinical prognostic factors and provide additional insights into treatment response and patient outcomes. Integrating genomic biomarkers such as Neo-P with clinical parameters could enhance prognostic stratification and inform personalized treatment strategies in TNBC.

Prognostic significance of a novel gene list did not stratify other datasets, such as GEO datasets. This may be due to two reasons: firstly, neoadjuvant therapy protocols differ in individual cohorts, secondly, the prognostic gene signature for breast cancer seems to be cohort specific. This second reason is discussed in greater detail in the analysis reported by Akbar et al. [11].

USAT results generated independently from two other cohorts did not show overlapping genes, so no further GSEA testing was performed. The authors note that further statistical research could be considered to establish “functional overlap” for each dataset USAT results, even if the genes themselves does not overlap.

Furthermore, the study investigated the predictive potential of a gene signature (NS/E vs. CS/M) for chemotherapy sensitivity. The observed shift towards a more mesenchymal phenotype following chemotherapy underscores the dynamic nature of TNBC tumors and highlights the complex interplay between tumor biology and treatment response. In our study, chemotherapy treatment resulted in a shift from the epithelial to the mesenchymal type. The lack of significance is most likely due to the small study sample. These findings underscore the need for personalized treatment approaches to address inherent heterogeneity and treatment resistance in TNBC.

Additionally, the study explored the plasticity of PAM50 subtypes in response to chemotherapy and during metastasis. Significant changes in PAM50 subtypes following chemotherapy suggest treatment-induced alterations in tumor characteristics. Similarly, metastatic samples exhibited distinct PAM50 subtypes compared to primary tissues, indicating a potential switch in tumor phenotypes during metastatic progression. These findings highlight the dynamic nature of TNBC subtypes and underscore the importance of re-evaluating subtyping approaches in residual cancer and metastatic tissues [11,14,15,16].

Overall, the study provides valuable insights into the molecular heterogeneity and dynamic nature of TNBC, emphasizing the importance of identifying prognostic and predictive biomarkers for personalized treatment approaches. Integrating genomic biomarkers such as Neo-P with clinical parameters could enhance prognostic stratification and inform tailored treatment strategies in TNBC. Further research is warranted to elucidate the underlying mechanisms driving treatment resistance and subtype plasticity, ultimately leading to improved patient outcomes in TNBC.

## 4. Methods and Materials

### 4.1. Sample Collection

Twenty-two samples of TNBC in the form of formalin fixed and paraffin embedded (FFPE) histopathological blocks were collected. Most of those samples were collected during cancer removal operation, but some were collected during cancer diagnosis process via core needle biopsy procedure. Patient data have been included in the Supplementary Materials (Supplementary Table S9). The sources of samples were Military Institute of Medicine—National Research Institute and Warmian-Masurian Cancer Center of the Ministry of the Interior and Administration Hospital. Both of these institutions obtained consent from bioethics committees to share samples (55/WIM/2015 and WMIL-KB/345/2019).

### 4.2. RNA Isolation and Sequencing

FFPE blocks were cut using manual microtome machine. First, 2–3 sections of each block were discarded. Two sections with a thickness 4 µm were combined in one preparation. If the RNA yield and purity were deemed unsatisfactory during first isolation, then 3–4 sections with a thickness 4 µm were used. Extraction of mRNA was performed immediately after microdissection using the RNeasy FFPE Kit (Qiagen, Stockach, Germany) according to the manufacturer’s protocol, and the amounts of reagents were adjusted to the number of sections, according to the manufacturer’s recommendations. Briefly, sections were placed in microcentrifuge tubes of appropriate size and 1 mL of xylene per sample was added to deparaffinize them. Then, vortexing was performed for 10 s and the sample was centrifuged. The supernatant was removed and 1 mL of 96% ethanol was added, vortexed and centrifuged. The ethanol was removed using a pipette and the open tube was left at room temperature until the remaining ethanol evaporated completely (approximately 10–15 min). Then, PKD buffer was added to the tube, mixed and vortexed. Proteinase K was also added by incubation at 56 °C for 15 min and then 80 °C for 15 min. The tube was incubated on ice for 3 min and then centrifuged. The supernatant was transferred to a new tube and DNase Booster Buffer and DNase I stock solution were added to it. The sample was gently mixed and centrifuged and then incubated at room temperature for 15 min. RBC buffer was added to the sample and the sample was thoroughly mixed. Next, 100% ethanol was added to the sample. The mixture was then transferred to a spin column and the flow-through was discarded after each spin until all of the original sample had passed through the column. The column was then washed twice with buffer and the flow-through was discarded. The column was placed in a new tube, the column lid was opened, and the column was centrifuged at full speed for 5 min. The column was placed in a new tube, 14 µL of RNase-free water was added, and the column was centrifuged. The final eluate yield was approximately 12 μL. RNA quantity and quality were assessed by UV absorption on a NanoDrop 1000 spectrophotometer, and fragment size was determined using the RNA 6000 Nano assay (Agilent Technologies, Santa Clara, CA, United States of America) on the 2100 Bioanalyzer. The percentage of RNA fragments above 200 nucleotides (DV200 value) was analyzed according to manufacturer’s instruction. Sequencing was performed by an external company. RNA library was prepared using 10 ng of material by Smart-Seq Stranded Kit (Takara, Shiga, Japan). The Q30 value was >94%. The sequencing parameters were 2 × 100 bp. Demultiplexing of the sequencing reads was performed with Illumina bcl2fastq (2.20). Adapters were trimmed with Skewer (version 0.2.2) [17]. The quality of the FASTQ files was analyzed with FastQC (version 0.11.5-cegat) [18].

### 4.3. cDNA Generation and qPCR Analysis

Reverse transcription was performed using the High-Capacity RNA-to-cDNA Kit (Applied Biosystems, Foster City, CA, United States of America) according to the manufacturer’s protocol, using a maximum of 2 μg of RNA per reaction. Briefly, the kit components were thawed on ice and appropriate amounts of RT reaction mix were aliquoted on a plate. The plates were closed with an appropriate seal. The plates were centrifuged to remove air bubbles. Plates were kept on ice until the PCR reaction was initiated. Plates were incubated for 60 min at 37 °C. The reaction was stopped by heating to 95 °C for 5 min and then holding at 4 °C. Real-time PCR was performed using iTaq Universal SYBR Green Supermix (Bio-rad, Hercules, CA, United States of America). First, all reaction components were thawed on ice. Then, all ingredients except the DNA template were mixed on a plate placed on ice. The reaction mix was mixed carefully. DNA samples were added, and the plate was sealed with optically transparent film and vortexed for 30 s. The plates were vortexed to remove air bubbles and place the reaction mixture at the bottom of the wells. Thermal cycling protocol on a real-time PCR instrument (AriaDx Real-Time PCR System Agilent Technologies) consisted of 30 s of polymerase activation and DNA denaturation at 95 °C and 40 cycles consisting of 15 s of denaturation at 95 °C and 60 s of annealing at 60 °C.

### 4.4. Analysis of Publicly Available Datasets

For in silico gene expression analysis, microarray datasets were downloaded from genomic data hosting websites, ArrayExpress (https://www.ebi.ac.uk/arrayexpress/ (accessed on 14 February 2023)) and Gene expression Omnibus (GEO, http://www.ncbi.nlm.nih.gov/geo/ (accessed on 10 March 2023)). Each dataset was RMA-normalized using BRB array tools [19]. Cluster tree 3.0 program [20] was used to hierarchically cluster data and heatmaps were generated using Java Treeview [21]. For both genes and samples, Euclidean distances were calculated using complete linkage.

### 4.5. Identifying a Prognostic Signature for TNBC Patients

An in-house R-based script (Unsupervised Survival Analysis Tool, i.e., USAT) performing three statistical tests: Cox proportional hazards regression (R{survival}) [22], maximally selected rank statistics (R{maxstat}) [23,24] and log-rank (R{survival}) test [25], the latter used to generate a prognosis related gene list. The R script used in this manuscript is included in the Supplementary Materials (Supplementary Text S1). Overall survival, from the date of diagnosis, was used as the end-point measure. The significant genes with a *p* value of lower than 0.05 in all three statistical tests were selected. The up/down-regulated genes were determined based on the hazard regression coefficient values where the genes with coefficient values greater than 1 are upregulated in a bad-survival group, while the genes with coefficient values of less than 1 are down-regulated in the bad-survival group. The thresholds for high and low expression of the genes were also calculated by the Maxstat test; therefore, each gene has different cut-off values based on Maxstat results.

### 4.6. Determining Gene Expression Differences in Samples from before and after Chemotherapy

To evaluate changes in tumor gene expression, we determined the “stemness score” of tumors using a 15-gene signature (CNCL: cancer stem/non-stem gene list) that contains genes related to EMT and stemness, as described before [11]. The stemness scores were calculated by generating correlation values of each sample to two matrices generated from the CNCL gene list (NS/E: non-stem, epithelial; CS/M: stem, mesenchymal) and subsequently obtaining the difference (Delta (r)) of the two (i.e., CS/M (r)—NS/E (r)), with negative values indicating a more NS/E phenotype [11]. Details on Delta(r) calculations are provided in the Supplementary Materials (Supplementary Text S2).

### 4.7. PAM50 Plasticity

Datasets GSE10281, GSE32518, GSE32072 (patient samples before and after treatment), GSE32518 (patient samples collected with fine needle aspiration and core needle biopsy), GSE125989 and GSE110590 (patient samples for primary and metastatic tissues) were downloaded from GEO NCBI database. Data regarding the datasets used in this publication are included in the Supplementary Materials (Supplementary Table S10). All datasets were “justRMA” normalized using BRB Array Tools [19]. PAM50 signature was used to classify breast cancer into 5 molecular sub-groups, namely Luminal A (LumA), Luminal B (LumB), HER2 enriched (HER2), basal and normal-like (Normal), as described previously [26] for each dataset. Paired samples were matched for pre- and post-therapy, with fine needle aspiration and core needle biopsy, or primary and metastatic tissue to compare PAM50 plasticity in these samples.

### 4.8. Statistical Analysis

Different treatment groups were compared using “*t*-test” and graphs were generated using GraphPad Prism v 6.01 (GraphPad Software Inc., New York City, NY, USA) and CorelDRAW 2017 (Corel Corporation, Ottawa, ON, Canada). For survival analysis, Cox proportional hazard regression and Log-Rank tests were performed using the “Survival” package in R [22,25]. Kaplan–Meier analysis was performed using IBM SPSS Statistics v.19.

## 5. Conclusions

In conclusion, our study represents a significant advancement in the understanding and management of triple-negative breast cancer (TNBC), a subtype known for its aggressive nature and limited treatment options. Through the identification of a novel gene list (Neo-P) derived from RNA sequencing data, we have successfully unveiled biomarkers that hold promise in predicting the prognosis of TNBC patients eligible for neoadjuvant therapy. This breakthrough not only enhances our ability to accurately subtype TNBC but also opens avenues for more targeted and effective treatment strategies.

Our analysis has further shed light on the plasticity of PAM50 subtypes in response to chemotherapy and metastasis. We observed significant subtype switching, both following chemotherapy treatment and during metastatic progression, underscoring the dynamic nature of TNBC subtypes. Importantly, our findings highlight the potential transformation of tumors from an epithelial to a more mesenchymal phenotype following chemotherapy, which may be associated with poorer prognosis and reduced treatment response. As such, our study emphasizes the need for re-evaluating subtyping approaches for residual cancer or metastasis, ensuring accurate classification for tailored treatment regimens.

Furthermore, our investigation revealed insights into the stability of different PAM50 subtypes, with the basal phenotype demonstrating the most stability and LumA being the most unstable. Additionally, we identified the influence of sampling methods on PAM50 classification, emphasizing the importance of standardized protocols to ensure consistent and accurate subtype classification.

Overall, our study underscores the importance of integrating genomic biomarkers with clinical parameters to enhance prognostic stratification and inform personalized treatment approaches in TNBC. By unraveling the intricate molecular mechanisms underlying treatment response and subtype plasticity, we pave the way for the development of more effective therapeutic interventions and improved patient outcomes in TNBC. Moving forward, further research is warranted to elucidate the underlying mechanisms driving treatment resistance and subtype plasticity, ultimately advancing the field towards more personalized and targeted approaches in TNBC management.

## Figures and Tables

**Table 1:** Spearman correlation of qPCR analysis results with RNAseq, normalized based on DESeq, FPKM or TMM.

Spearman Rho Values				qPCR Data (-ddCT)				DESeq2 Normalized RNA-seq Data				FPKM of RNA-seq Data				TMM Normalized			
Gene Name	GDSC1	GDSC2	CTD		Mean BC	Mean AC	*T*-Test P		Mean BC	Mean AC	*T*-Test P		Mean BC	Mean AC	*T*-Test P		Mean BC	Mean AC	*T*-Test P
ABHD4	0.48	0.51	0.32	1	6.37	2.98	0.00	0	7.67	7.98	0.08	0	0.93	1.37	0.00	0	7.55	8.12	0.00
CDYL2	0.89	0.34	0.43	1	8.35	7.39	0.45	1	8.28	8.24	0.85	0	0.74	0.94	0.04	0	8.15	8.38	0.27
COPS7A	0.45	0.56	0.38	1	7.72	4.23	0.00	1	8.66	8.61	0.73	0	1.75	2.05	0.07	0	8.53	8.75	0.25
DPM2	0.79	0.54	0.38	1	11.64	8.75	0.00	1	7.89	7.68	0.18	0	1.26	1.42	0.23	0	7.75	7.82	0.77
GSG1	0.32	0.35	0.34	1	10.74	7.88	0.01	1	2.30	1.11	0.11	1	0.11	0.02	0.06	1	2.24	0.98	0.05
JMJD7	−0.35	−0.47	−0.32	0	10.78	7.17	0.00	1	5.13	5.33	0.30	1	0.43	0.59	0.00	1	5.07	5.47	0.04
KIAA1456	0.39	0.34	0.50	1	11.04	6.33	0.00	1	7.54	7.39	0.74	0	0.41	0.44	0.80	0	7.41	7.53	0.78
KRTAP 17-1	−0.48	−0.35	−0.32	0	12.07	6.47	0.00	0	1.05	0.36	0.13	0	0.07	0.02	0.29	0	0.92	0.42	0.20
LIN9	−0.36	−0.37	−0.35	0	9.49	7.21	0.02	0	7.83	7.40	0.09	1	1.00	1.03	0.83	0	7.71	7.54	0.53
MIGLL	0.47	0.48	0.42	1	6.27	4.59	0.00	0	8.67	9.09	0.10	0	0.79	1.28	0.00	0	8.54	9.23	0.00
OFD1	0.82	0.36	0.45	1	6.44	4.19	0.00	0	10.64	10.76	0.50	0	2.72	3.23	0.04	0	10.51	10.90	0.11
PHKA2	0.82	0.35	0.40	1	8.25	6.05	0.01	1	9.36	9.24	0.27	0	1.20	1.43	0.04	0	9.23	9.38	0.26
PRMT7	0.82	0.36	0.48	1	10.93	6.34	0.00	1	9.31	9.24	0.55	0	1.12	1.38	0.01	0	9.17	9.38	0.07
ST3GAL4	0.37	0.34	0.31	1	7.12	3.31	0.00	1	8.91	8.77	0.52	0	1.57	1.80	0.21	0	8.78	8.91	0.58
TAL2	−0.37	−0.44	−0.34	0	9.09	3.64	0.00	0	1.76	0.87	0.11	0	0.15	0.08	0.15	0	1.62	0.96	0.14
TMTM221	0.85	0.35	0.36	1	7.64	3.50	0.00	1	2.35	1.53	0.14	1	0.08	0.05	0.20	1	2.16	1.51	0.16
ZDHHC7	0.42	0.37	0.40	1	6.12	3.41	0.00	0	9.35	9.51	0.27	0	2.03	2.49	0.01	0	9.22	9.65	0.05

**Table 2:** Comparison of PAM50 subtypes pre- and post-Letrozole therapy (GSE10281).

		Post-Therapy				
		Basal	HER2	LumA	LumB	Normal
Pre-therapy	Basal	2	0	0	0	0
	HER2	1	1	0	2	0
	LumA	0	0	2	3	0
	LumB	0	0	0	1	0
	Normal	3	0	1	1	1

**Table 3:** Comparison of PAM50 subtypes pre- and post-anthracycline-based and taxane-based neoadjuvant chemotherapy (GSE32518).

		Post-Therapy				
		Basal	HER2	LumA	LumB	Normal
Pre-therapy	Basal	2	0	0	0	2
	HER2	1	1	0	1	1
	LumA	0	0	3	0	4
	LumB	1	0	4	3	3
	Normal	0	0	1	0	1

**Table 4:** Comparison of PAM50 subtypes pre- and post-neoadjuvant chemotherapy (GSE32072).

		Post-Therapy				
		Basal	HER2	LumA	LumB	Normal
Pre-therapy	Basal	5	0	0	0	0
	HER2	0	4	0	0	0
	LumA	0	0	5	1	0
	LumB	0	0	0	1	0
	Normal	2	1	1	0	1

**Table 5:** Comparison of PAM50 subtyping for primary tissues vs. brain metastatic tissues (GSE125989).

		Metastasized Tissue				
		Basal	HER2	LumA	LumB	Normal
Primary tissue	Basal	2	1	0	0	0
	HER2	1	2	0	0	1
	LumA	0	0	4	0	0
	LumB	0	0	0	0	0
	Normal	3	2	0	0	0

**Table 6:** Comparison of PAM50 subtyping for primary tissues vs. metastatic tissues (GSE110590).

		PAM50 Metastasis				
Sample Name	Primary Tissue	Basal	HER2	LumA	LumB	Normal
A11	Basal	5	0	0	0	0
A15	Basal	2	0	0	0	3
A1	Basal	4	0	0	0	1
A23	Basal	5	0	0	0	0
A5	Basal	2	0	0	0	0
A7	Basal	5	0	0	0	0
A8	HER2	0	4	0	0	0
A12	LumA	0	0	2	4	0
A17	LumA	2	0	0	0	0
A26	LumA	3	0	0	0	0
A28	LumA	0	0	3	3	0
A2	LumA	0	1	0	2	0
A30	LumA	5	0	0	0	0
A34	LumA	0	0	4	0	0
A4	LumA	0	1	1	0	0
A20	Normal	5	0	0	0	0

## Data Availability

The data presented in this study are available in in the article and in the Supplementary Materials. Some of the data regarding sequenced RNA from patients presented in this study are available on request from the corresponding author. The data resulting from the RNA sequencing of patients will be made available after the publication of works related to this material.

## Supplementary Materials

The supporting information can be downloaded at: https://www.mdpi.com/article/10.3390/ijms25116054/s1.
