# Supplementary material for: Breast Cancer Plasticity after Chemotherapy Highlights the Need for Re-Evaluation of Subtyping in Residual Cancer and Metastatic Tissues
Source: Int J Mol Sci. 2024 May 31;25(11):6054. doi: 10.3390/ijms25116054 (PMC11172877; doi:10.3390/ijms25116054)
Supplement: Supplementary file 1 [file ijms-25-06054-s001.zip › Table S3 A list of significant prognostic factors.pdf]

Supplementary Table S3. A list of significant prognostic factors

**Progression status**		Frequency	Percent	Valid Percent	Cumulative Percent
Valid	0	12	50	50	50
	1	12	50	50	100
	Total	24	100	100	

Omnibus Tests of Model Coefficients

-2 Log Likelihood	Overall (score)			Change From Previous Step			Change From Previous Block		
	Chi-square	df	Sig.	Chi-square	df	Sig.	Chi-square	df	Sig.
41.255	10.489	1	0.001	10.863	1	0.001	10.863	1	0.001

**Radiological regression**		Frequency	Percent	Valid Percent	Cumulative Percent
Valid	0	12	50	50	50
	1	12	50	50	100
	Total	24	100	100	

Variables in the Equation

						95.0% Cl for Exp(B)	
B	SE	Wald	df	Sig.	Exp(B)	Lower	Upper
-2.524	1.065	5.617	1	0.018	0.080	0.010	0.646

**Clinical regression**		Frequency	Percent	Valid Percent	Cumulative Percent
Valid	0	9	37.5	37.5	37.5
	1	15	62.5	62.5	100
	Total	24	100	100	

Variables in the Equation

						95.0% Cl for Exp(B)	
B	SE	Wald	df	Sig.	Exp(B)	Lower	Upper
-2.226	0.0806	7.621	1	0.006	1.08	0.022	0.524

Relapse		Frequency	Percent	Valid Percent	Cumulative Percent
Valid	0	12	50	50	50
	1	12	50	50	100
	Total	24	100	100	

Variables in the Equation							
						95.0% Cl for Exp(B)	
B	SE	Wald	df	Sig.	Exp(B)	Lower	Upper
2.732	1.083	6.360	1	0.012	15.367	1.838	128.427
