# Supplementary material for: Breast Cancer Plasticity after Chemotherapy Highlights the Need for Re-Evaluation of Subtyping in Residual Cancer and Metastatic Tissues
Source: Int J Mol Sci. 2024 May 31;25(11):6054. doi: 10.3390/ijms25116054 (PMC11172877; doi:10.3390/ijms25116054)
Supplement: Supplementary file 1 [file ijms-25-06054-s001.zip › Table S4 A list of not significant prognostic factors.pdf]

Table S4. Univariate analyses of clinical confounding factors

Localization of Tumor (quadrant)*

		Frequency	Percent	Valid percent	Cumulative percent
Valid	2	9	37,5	37,5	37,5
	3	8	33,3	33,3	70,8
	4	7	29,2	29,2	100,0
	Total	24	100,0	100,0	

Variables in the Equation

						95,0,% CI for Exp(B)	
B	SE	Wald	df	Sig.	Exp(B)	Lower	Upper
-0,130	0,401	0,106	1	0,745	0,878	0,400	1,926

*Quadrant upper right=1, Lower right=2, Upper left=3, Lower left=4, central=5

Localization of Tumor (side)**

		Frequency	Percent	Valid percent	Cumulative percent
Valid	1	16	66,7	66,7	66,7
	2	8	33,3	33,3	100,0
	Total	24	100,0	100,0	

Variables in the Equation

						95,0,% CI for Exp(B)	
B	SE	Wald	df	Sig.	Exp(B)	Lower	Upper
-0,029	0,709	0,002	1	0,968	0,972	0,242	3,897

**Side Left=1, Right=2

Biopsy type***

		Frequency	Percent	Valid percent	Cumulative percent
Valid	1	3	12,5	12,5	12,5
	2	21	87,5	87,5	100,0
	Total	24	100,0	100,0	

Variables in the Equation

						95,0,% CI for Exp(B)	

B	SE	Wald	df	Sig.	Exp(B)	Lower	Upper
-0,711	0,818	0,757	1	0,384	0,491	0,099	2,439

***Fine needle biopsy=1, core needle biopsy=2

Grade

		Frequency	Percent	Valid percent	Cumulative percent
Valid	2	4	16,7	16,7	16,7
	3	20	83,3	83,3	100,0
	Total	24	100,0	100,0	

Variables in the Equation

						95,0,% CI for Exp(B)	
B	SE	Wald	df	Sig.	Exp(B)	Lower	Upper
-0,449	0,811	0,307	1	0,580	0,638	0,130	3,127

Pathological response****

		Frequency	Percent	Valid percent	Cumulative percent
Valid	0	1	4,2	4,2	4,2
	1	5	20,8	20,8	25,0
	2	18	75,0	75,0	100,00
	Total	24	100,0	100,0	

Variables in the Equation

						95,0,% CI for Exp(B)	
B	SE	Wald	df	Sig.	Exp(B)	Lower	Upper
3,054	2,512	1,479	1	0,224	21,203	0,154	2913,208

****Pathological complete response=1, Residual Disease=2, pathological response not assessed = 0

AJCC Staging

		Frequency	Percent	Valid percent	Cumulative percent
Valid	2a	3	12,5	12,5	12,5
	2b	10	41,7	41,7	54,2
	3a	6	25,0	25,0	79,2
	3b	5	10,8	10,8	100,0
	Total	24	100,0	100,0	

Variables in the Equation

	B	SE	Wald	df	Sig.	Exp(B)	95,0,% CI for Exp(B)	
							Lower	Upper
AJCC			6,570	3	0,087			
AJCC(1)	-1,434	1,133	1,601	1	0,206	0,238	0,026	2,196
AJCC(2)	-2,805	1,150	5,950	1	0,015	0,060	0,006	0,576
AJCC(3)	-1,026	0,840	1,491	1	0,222	0,358	0,069	1,860

TNM Staging – T grade

		Frequency	Percent	Valid percent	Cumulative percent
Valid	2	11	45,8	45,8	45,8
	3	8	33,3	33,3	79,2
	4	5	20,8	20,8	100,0
	Total	24	100,0	100,0	

Variables in the Equation

	B	SE	Wald	df	Sig.	Exp(B)	95,0,% CI for Exp(B)	
							Lower	Upper
T	0,840	0,465	3,265	1	0,071	2,316	0,931	5,760

TNM Staging – N grade

		Frequency	Percent	Valid percent	Cumulative percent
Valid	0	6	25,0	25,0	25,0
	1	12	50,0	50,0	75,0
	2	5	20,8	20,8	95,8
	3	1	4,2	4,2	100,0
	Total	24	100,0	100,0	

Variables in the Equation

	B	SE	Wald	df	Sig.	Exp(B)	95,0,% CI for Exp(B)	
							Lower	Upper
N	0,309	0,386	0,642	1	0,423	1,362	0,640	2,902
