# Supplementary material for: Breast Cancer Plasticity after Chemotherapy Highlights the Need for Re-Evaluation of Subtyping in Residual Cancer and Metastatic Tissues
Source: Int J Mol Sci. 2024 May 31;25(11):6054. doi: 10.3390/ijms25116054 (PMC11172877; doi:10.3390/ijms25116054)
Supplement: Supplementary file 1 [file ijms-25-06054-s001.zip › Table S5 A list of significantly correlated parameters.pdf]

Supplementary Table S5. A list of significantly correlated parameters

		Radiological Regression			
		0	1	Total	
Progression status	0	2	10	12	
	1	10	2	12	
Total		12	12	24	
Symmetric Measures					
		Value	Asymptotic Standard Error[1]	Approximate T[2]	Approximate Significance[3]
Interval by Interval	Pearson's R	-0.667	-0.152	-4.195	0.000
Ordinal by Ordinal	Spearman Correlation	-0.667	-0.152	-4.195	0.000
N of Valid Cases		24			
		Clinical Regression			
		0	1	Total	
Progression status	0	1	11	12	
	1	8	4	12	
Total		9	15	24	
Symmetric Measures					
		Value	Asymptotic Standard Error[1]	Approximate T[2]	Approximate Significance[3]
Interval by Interval	Pearson's R	-0.602	-0.153	-3.540	0.002

Ordinal by Ordinal	Spearman Correlation	-0.602	-0.153	-3.540	0.002
N of Valid Cases		24			

		Relapse		
		0	1	Total
Progression status	0	12	0	12
	1	0	12	12
Total		12	12	24

Symmetric Measures

		Value	Asymptotic Standard Error[45]
Interval by Interval	Pearson's R	1.000	0.000
Ordinal by Ordinal	Spearman Correlation	1.000	0.000
N of Valid Cases		24	
