# Supplementary material for: Breast Cancer Plasticity after Chemotherapy Highlights the Need for Re-Evaluation of Subtyping in Residual Cancer and Metastatic Tissues
Source: Int J Mol Sci. 2024 May 31;25(11):6054. doi: 10.3390/ijms25116054 (PMC11172877; doi:10.3390/ijms25116054)
Supplement: Supplementary file 1 [file ijms-25-06054-s001.zip › Table S6 Confounding factor analyses.pdf]

Supplementary Table S6. Confounding factor analysis

Backward Wald

Variables in the Equation[6]									
								95.0% CI for Exp(B)	
		B	SE	Wald	df	Sig.	Exp(B)	Lower	Upper
Step 1	Relapse, 0 for no and 1 for yes	1.765	1.239	2.029	1	0.154	5.841	0.515	66.263
	Progression status				0[7]				
	Clinical regression	-0.902	1.085	0.691	1	0.406	0.406	0.048	3.404
	Radiological regression	-0.789	1.519	0.270	1	0.604	0.454	0.023	8.923
Step 4	Relapse, 0 for no and 1 for yes	2.732	1.083	6.360	1	0.012	15.367	1.838	128.427

Forward Wald

Variables in the Equation[8]									
								95.0% CI for Exp(B)	
		B	SE	Wald	df	Sig.	Exp(B)	Lower	Upper
Step 1	Clinical regression	-2.226	0.806	7.621	1	0.006	0.108	0.022	0.524

Variables not in the Equation				
		Score	df	Sig.
Step 1	Relapse, 0 for no and 1 for yes	3.072	1	0.080
	Progression status	3.072	1	0.080
	Radiological regression	1.640	1	0.200
