# Supplementary material for: Breast Cancer Plasticity after Chemotherapy Highlights the Need for Re-Evaluation of Subtyping in Residual Cancer and Metastatic Tissues
Source: Int J Mol Sci. 2024 May 31;25(11):6054. doi: 10.3390/ijms25116054 (PMC11172877; doi:10.3390/ijms25116054)
Supplement: Supplementary file 1 [file ijms-25-06054-s001.zip › Table S7 Multivariate model analysis.pdf]

Supplementary Table S7. Multivariate model analysis in 24 patients

Variables in the Equation								
							95.0% CI for Exp(B)	
	B	SE	Wald	df	Sig.	Exp(B)	Lower	Upper
Clinical regression	-0.225	0.851	0.070	1	0.791	0.798	0.151	4.229
USAT defined prognostic groups	12.831	123.686	0.011	1	0.917	373657.936	0.000	7.152E+110
