# Supplementary material for: Breast Cancer Plasticity after Chemotherapy Highlights the Need for Re-Evaluation of Subtyping in Residual Cancer and Metastatic Tissues
Source: Int J Mol Sci. 2024 May 31;25(11):6054. doi: 10.3390/ijms25116054 (PMC11172877; doi:10.3390/ijms25116054)
Supplement: Supplementary file 1 [file ijms-25-06054-s001.zip › Text S2 Calculation of Delta r.pdf]

Calculation of Delta(r)

Delta(r) is a measure that indicates the epithelial to mesenchymal transition state of samples by using a set of gene markers. It is calculated as follows.

- We have a pattern of the marker genes published in our previous study. This pattern is the relative expression weights of the genes. Positive values represent high expression, negative values represent low expression. The pattern values are as follows:

Gene symbol/Probeset	Group	CS/M	NS/E
DDR2\U\205168_at	CS/M	1.87	-0.507
DKK3\U\202196_s_at	CS/M	1.866	-0.524
SLIT2\U\209897_s_at	CS/M	1.698	-0.588
PVRL3\U\213325_at	CS/M	1.601	-0.7
BNC2\U\220272_at	CS/M	1.544	-0.662
TMEM158\U\213338_at	CS/M	1.534	-0.689
FN1\U\212464_s_at	CS/M	1.498	-0.312
VIM\U\201426_s_at	CS/M	1.351	-0.815
ST14\D\202005_at	NS/E	-1.447	0.544
BSPRY\D\218792_s_at	NS/E	-1.539	0.625
ZNF165\D\206683_at	NS/E	-1.566	0.537
CLDN4\D\201428_at	NS/E	-1.572	0.515
CDH1\D\201131_s_at	NS/E	-1.672	0.717
AP1M2\D\65517_at	NS/E	-1.807	0.518

- To calculate the Delta(r) of a sample, first E(r) and M(r) values were calculated as the correlation between the expression of the marker genes in the sample and the pattern values of the marker genes (E for Epithelial and M for Mesenchymal).

- Delta(r) value is the difference between M(r) and E(r) values such that Delta(r) = M(r) – E(r).

- **Interpretation:** If the expression of the marker genes is more correlated with Mesenchymal pattern values, than M(r) value will be higher than the E(r) value, thus leading to a positive Delta(r) value. In this case the sample will be more mesenchymal. Otherwise, the sample will be more epithelial (negative Delta(r) value) if the correlation between the expression of the marker genes is more correlated with the epithelial pattern values.
